# Initial experience with MR-guided adaptive spinal stereotactic radiotherapy: a new indication for the MR-linac

**DOI:** 10.1007/s00066-025-02401-3

**Published:** 2025-04-28

**Authors:** Neris Dincer, Teuta Zoto Mustafayev, Ceren Atahan, Gorkem Gungor, Gamze Ugurluer, Mehmet Ufuk Abacioglu, Enis Ozyar, Banu Atalar

**Affiliations:** 1 Department of Radiation Oncology, Acıbadem MAA University School of Medicine, 34450 Istanbul, Turkey; 2 Department of Radiation Oncology, Acıbadem Maslak Hospital, Istanbul, Turkey

**Keywords:** Spinal metastases, MR-linac, SMART, Spinal cord toxicity, Online adaptive planning

## Abstract

**Background and purpose:**

Stereotactic body radiotherapy (SBRT) is associated with good local control and symptom relief in the management of spinal metastases. Delivery of ablative doses and re-irradiation is challenged by spinal cord toxicity. We hypothesized that lower spinal cord doses as well as better target coverage could be yielded with stereotactic magnetic resonance-guided adaptive radiotherapy (SMART).

**Materials and methods:**

Institutional records were reviewed to retrieve patients who received online MR-guided SBRT for spinal metastases. Each fraction was reviewed to determine the necessity of adaptive planning, to identify reasons for violations that required adaptive planning, and to assess the spinal cord dose. The study also evaluated how adaptive planning contributed to reducing spinal cord doses.

**Results:**

A total of 34 patients with 61 lesions were included. The treatment intent was definitive for 47 (77.1%), palliative for 12 (19.7%), and postoperative for two (3.3%) lesions. The median prescribed Biological Equivalent Dose (BED)_10_ was 51.3 Gy. Treatment plans often required adaptive adjustments (81.8%). Adaptive planning significantly improved target coverage (median PTV coverage 92.75% vs. 95%; *p* < 0.001) and reduced spinal cord D_max_ (median spinal cord D_max_ constraint: 7.3 Gy, median predicted spinal cord D_max_: 7.76, and median adaptive spinal cord D_max_ 6.18; *p* < 0.001). Lesion-based median follow-up from irradiation was 7.5 months (range: 1–46 months). One-year LPFS was 94.3%. Six lesions progressed and none of the progressed lesions received a dose above the median BED_10_ of 51.3 Gy.

**Conclusion:**

Herein we present our institutional experience with SMART for spinal bone metastases. According to our results, adaptive planning yields better target coverage as well as lower spinal cord doses compared to the predicted plan, which translates into a feasible method for delivering SBRT. Future prospective studies evaluating spinal SMART are awaited.

## Introduction

The spine is the most common site of bone metastasis and spinal metastases affect nearly 30% of metastatic cancer patients [1]. Spinal metastases may be associated with serious symptoms, namely severe pain, fracture, and cord compression, necessitating local treatment of the metastases along with systemic treatment [2]. Stereotactic body radiotherapy (SBRT) enables delivery of a high biologically effective dose (BED) to the tumor, proving to be an efficient treatment method for symptom relief as well as durable local control and increased survival in oligometastatic disease [3] compared to conventional palliative radiotherapy schemes.

In the setting of oligometastatic disease, the treatment intent has shifted from palliation to ablation of oligometastases, as this approach was shown to be associated with improved overall survival by the SABR-COMET trial [4]. In this context, dose escalation has been investigated and found to be associated with improved local control [5]. However, delivery of ablative doses is challenged by spinal cord toxicity, which is further complicated by the difficulty of accurate visualization of the spinal cord in treatment plans of computer tomography (CT)-based linear accelerators (linacs). In clinical practice, various methods are used to overcome this problem. While delineating the spinal canal as a surrogate protects the true spinal cord, it also limits target coverage and poses a risk of missing the tumor. Alternatively, magnetic resonance image (MRI) acquisition and fusion with CT images is also used for spinal cord delineation. However, even with the optimal fusion, spinal cord movement during treatment may lead to detrimental toxicity [6].

Stereotactic MR-guided adaptive radiotherapy (SMART) has the advantage of onboard MRI acquisition, allowing online daily spinal cord delineation and daily plan adaptation [7]. We hypothesized that lower spinal cord doses as well as better target coverage could be yielded with SMART.

## Materials and methods

Ethical approval for this study was obtained from our institutional review board (date and protocol number: 09.01.2020 and 2020-01/4). The departmental database was reviewed to determine patients treated between September 2018 and October 2023 eligible for this retrospective study. Inclusion criteria were 1) SBRT for spinal metastasis using an MR-linac and 2) follow up of at least 3 months. Baseline patient characteristics including age, gender, primary, histopathology, metastatic pattern, lesion location (cervical, thoracal, lumbar, sacral), and whether the lesion was symptomatic were recorded. Metastatic lesions had been confirmed either with positron-emission tomography/computerized tomography (PET/CT) or MRI before treatment.

**Fig. 1:**
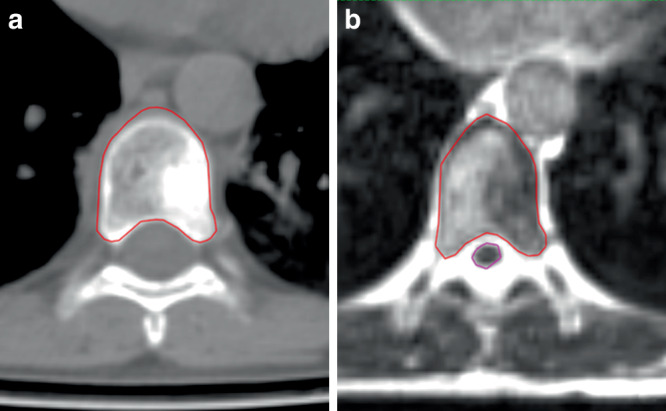
Delineation of CTVs and spinal cord on CT and MRI images. **a** Axial view of the CTV contour (red); **b** axial MRI slice of the CTV contour in red and spinal cord contour in violet. Note the spinal cord cannot be visualized on the CT image (**a**). *CTV* clinical target volume; *CT* computed tomography; *MRI* magnetic resonance imaging

All patients underwent a simulation MRI and CT scan; immobilization devices such as a thermoplastic mask, fiber glass wing board, or pelvic stabilizers were used, depending on the location of spinal metastasis. The gross tumor volume (GTV), clinical target volumes (CTVs), and organs at risks (OARs) were contoured on MR images (Fig. 1). CTV delineation was performed according to the International Spine Radiosurgery Consortium consensus guidelines [8] and was modified if the patient had a special consideration such as reirradiation of the vertebra or a previous radiotherapy field in close proximity. Planning target volumes (PTVs) were generated using margins depending on the target definition and surrounding organs: “When the PTV overlapped with the spinal cord, esophagus, sacral plexus, or other OARs, an alternative PTV (PTVopt) that did not intersect with these structures was created. The prescribed dose was delivered to PTVopt, and the goal was for 100% of the PTV (or PTVopt) to receive 95% of the prescribed dose. However, if this was not achievable due to OAR constraints, the coverage was reduced to 90%.”

A treatment plan was generated for each patient using the MRIdian (Viewray Inc., Oakwood Village, OH, USA) treatment planning system (version 5.5.5) that incorporates a 0.35‑T MR scanner and a linear accelerator delivering 6‑MV FFF photons. Before each fraction, targets and OARs were edited or recontoured by the treating physician. Recalculations were performed on baseline plan depending on the contours of that fraction, and a reoptimized plan was generated. Institutional dose constraints were used to evaluate target coverage and OAR doses (Table 1). Predicted plans and reoptimized plans were compared, and the delivery plan with the step-and-shoot IMRT technique was chosen. We tracked the tumor itself (vertebra), and the tolerance was 1% target out, the confidence level value was ≥ 80%, and the gating margin was 3 mm. To ensure that the spinal cord was visible, a sagittal plane well distinguishing the target and the spinal cord was chosen.

**Table 1:** Institutional dose constraints for single-, three-, and five-fraction SBRT

Structure	Single fraction	Three fractions	Five fractions
Spinal cord	D_max_ < 14 Gy	D_max_ < 21.9 Gy	D_max_ < 30 Gy
	≤ 10% at 10 Gy	≤ 10% at 18 Gy	≤ 10% at 23 Gy
	≤ 0.35 cc at 10 Gy	≤ 0.35 cc at 18 Gy	≤ 0.35 cc at 23 Gy
	≤ 1.2 cc at 7 Gy	≤ 1.2 cc at 12.3 Gy	≤ 1.2 cc at 14.5 Gy
	≤ 0.03 cc at 12.4 Gy	≤ 0.03 cc at 20.3 Gy	≤ 0.03 cc at 25.3 Gy
Cauda equina	≤ 5 cc at 14 Gy	≤ 5 cc 21.9 Gy	≤ 5 cc at 30 Gy
	≤ 0.03 cc at 16 Gy	≤ 0.03 cc at 24 Gy	≤ 0.03 cc at 32 Gy
Esophagus	D_max_ < 15.4 Gy	D_max_ < 25.2 Gy	D_max_ < 35 Gy
	≤ 5 cc at 11.9 Gy	≤ 5 cc at 17.7 Gy	≤ 5 cc at 19.5 Gy

*SBRT* stereotactic body radiotherapy; *D*_*max*_ maximum point dose to the organ

Patients’ follow-up medical records were reviewed to collect data on the clinical response, radiologic response (based on last control CT scan, MRI, or PET/CT), early and late adverse events as per the Common Terminology Criteria for Adverse Events (CTCAE) version 5.0, last control date, and last status of the patients. The reference date for the time-to-event analysis was defined as the last fraction of SMART. Local control (LC) was defined as the absence of progression of the same lesion. Responses were recorded as per initial control imaging and as per last control imaging. For fluorodeoxyglucose (FDG)- or prostate-specific membrane antigen labelled with 68 Ga (Ga-PSMA)-PET/CT follow-ups, a complete response was defined as disappearance of uptake, partial response was defined as a ≥ 30% decrease in maximum standard uptake value (SUV) compared to the previous imaging, and nonresponse/progression was defined as stable/increased SUV compared to the previous imaging [9]. Local progression-free survival (LPFS) was defined as time from the end of treatment to occurrence of event or last follow-up and was estimated using the Kaplan–Meier method and the log-rank test.

Dosimetric outputs were collected from the ViewRay MRIdian database. All SMART plans were examined and the total dose, number of fractions, and number of adapted fractions were recorded. Each fraction was individually assessed to generate the information regarding whether adaptive planning was generated and to determine the violation that necessitated adaptive planning, along with the spinal cord dose for each fraction. The impact of adaptive planning on the spinal cord dose reduction was analyzed. A *p*-value < 0.05 was considered statistically significant. Statistical analysis was performed using SPSS software version 23 (IBM, Armonk, NY, USA).

## Results

### Patient demographics and treatment characteristics

A total of 1172 lesions in 872 patients were treated with the MR-linac at our center between September 2018 and October 2023. The number of irradiated spinal bone metastases was 61 in 34 patients, with a median of one lesion per patient (range 1–5 lesions). The median prescribed dose was 24 Gy (range 18–50 Gy) in 3 (range 1–5) fractions. Doses were selected based on tumor size, relation to OARs, treatment intent, and whether the OARs had previously received radiation (exception: irradiation of a different vertebra) or whether irradiation was a reirradiation. Seven cases were reirradiation. Patient and treatment characteristics are listed in Table 2 and treatment planning output is listed in Table 3.

**Table 2:** Patient characteristics

Characteristic	Result
*Total no. of patients*	34 (100%)
*Total no. of lesions*	61
*Age (median)*	61.5 years (40–83)
*Gender (M/F)*	23/11
*Reirradiation*	7 patients (11.5%)
*Tumor location*	
Cervical	6 (9.8%)
Thoracal	31 (50.8%)
Lumbar	23 (37.7)
Sacral	1 (1.6%)
*Tumor location*	
Vertebral body	37 (60.7%)
Vertebral body + transverse process	11 (18.0%)
Transverse process	9 (14.8%)
Spinous process	4 (6.6%)
*Bilksy score*	
0	49 (80.3%)
1a	9 (14.8%)
1b	3 (4.9%)
*Treatment intent*	
Definitive (oligometastasis)	47 (77.1%)
Palliation	12 (19.7%)
Postoperative	2 (3.3%)

*GTV* gross tumor volume, *M* male, *F* female

**Table 3:** SMART treatment planning characteristics

Characteristic	Number (median, range)
*Prescribed dose (Gy)*	24 Gy (18–50 Gy)
*Number of fractions*	3 fractions (1–5 fractions)
1 fraction	4 (6.6%)
2 fractions	4 (6.6%)
3 fractions	45 (73.8%)
4 fractions	1 (1.6%)
5 fractions	7 (11.5%)
*Median BED*_*10*_* (Gy)*	51.3 Gy (28–100 Gy)
*BED*_*10*_* (Gy)*	
≤ 51.3	47
> 51.3	14
*Median GTV diameter (mm)*	24 mm (6–74 mm)
*Median GTV/CTV volume (cc)*	29.8 cc (5.8–794.5 cc)
*Median PTV volume (cc)*	36.6 cc (8.6–809.5 cc)
*PTV margin*	
No margin	13 (21.2%)
1 mm in all directions, 0 from posterior	9 (14.8%)
1 mm in all directions	19 (31.1%)
2 mm in all directions, 0 from posterior	1 (1.6%)
2 mm	6 (9.8%)
3 mm	13 (21.3%)
*Median treatment time (beam on; minutes)*	15.1 min (8.6–29.9 min)
*Median coverage compromise* *index (CCI)*	0.90 (0.64–0.98)
*PTV D*_*98%*_	94.2% (80.5–101%)
*PTV D*_*95%*_	100% (91.1–102.8%)
*PTV D*_*50%*_	112% (103.4–119.8%)
*PTV D*_*2%*_	118.9% (107.1–141.7%)

*PTV D*_*98*_* and D*_*2*_ minimum dose to 98% and 2% volumes, respectively; *PTV D*_*50*_ median dose, *PTV D*_*50*_ minimum dose delivered to 95% of the PTV; *GTV* gross tumor volume; *CTV* clinical tumor volume; *PTV* planning target volume; *SMART *stereotactic magnetic resonance-guided adaptive radiotherapy

### Target coverage and spinal cord

**Fig. 2:**
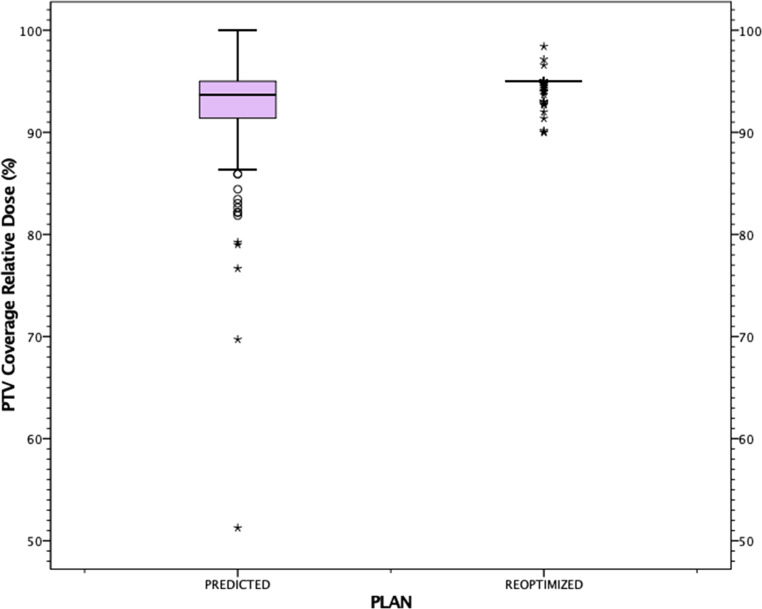
Planning target volume (*PTV*) dose coverage distributions on predicted and reoptimized plans

The total number of all fractions was 187. In 153 (81.8%) of the 187 fractions, treatments were performed with reoptimized plans. PTV coverage in the adapted plans was improved compared to the predicted plans (median PTVreopt 95% vs. median PTVpredict 92.75%; *p* < 0.001; Fig. 2).

**Fig. 3:**
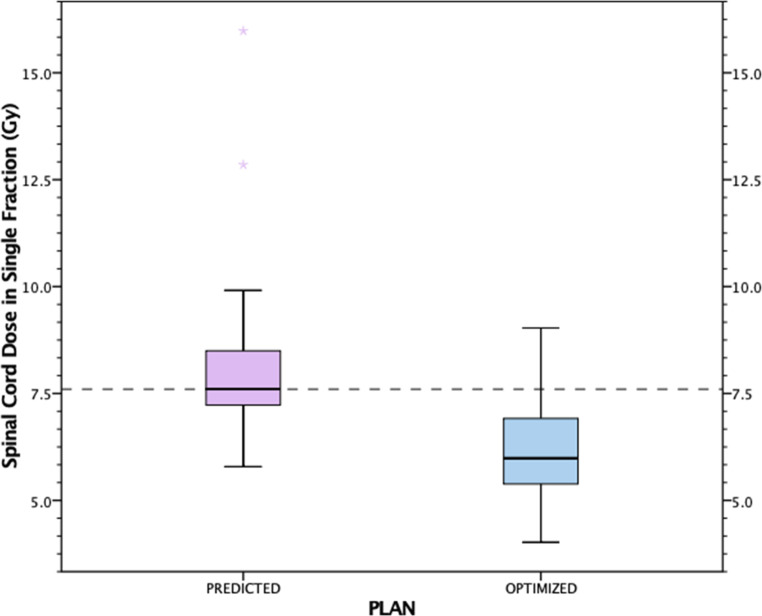
Predicted and reoptimized plan spinal cord dose on the violated fractions

The median spinal cord D_max_ in original plans was 12.02 Gy (10.29–13.66 Gy) for single-fraction (*n* = 4), 16.37 Gy (1.7–18.06 Gy) for two-fraction (*n* = 4), 16.20 Gy (0–21.03 Gy) for three-fraction (*n* = 45), 10.65 Gy for four-fraction (*n* = 1), and 14.16 Gy (0–21.24 Gy) for five-fraction (*n* = 7) treatments. In 24 (12.83%) of the predicted plans, spinal cord doses were violated, with a median spinal cord D_max_ constraint of 7.30 Gy per fraction, median predicted spinal cord D_max_ of 7.76 Gy, and median adaptive spinal cord D_max_ of 6.18 Gy (*p* < 0.001; Fig. 3).

### Local control

Lesion-based median follow-up from irradiation was 7.5 months (range 1–46 months). Symptom relief was achieved in all but two of the lesions. Median time from the last fraction of SMART to the first follow-up imaging was 2.6 months (range 0.6–19.1 months). One patient who was evaluated after 19.1 months was a prostate cancer patient who was followed with prostate-specific antigen (PSA) levels. As per first follow-up imaging, complete response was achieved in 24 (39.3%) lesions and partial response was achieved in 28 (45.9%) lesions; 3 lesions (4.3%) remained stable and 1 (1.4%) progressed, whereas patients with 5 (7.2%) lesions were lost to follow-up. Among 29 lesions that were re-evaluated with follow-up imaging, 18 of the complete responders remained in complete response, whereas 1 lesion progressed; 3 of the partial responders remained in partial response, 2 eventually became complete responders, and 3 lesions progressed. One of the stable lesions partially responded and one progressed. In sum, 6 lesions progressed.

One-year LPFS was 94.3%, and 2‑year LPFS was 73.6%. The influence of BED_10_ (above median vs. median and below median) on local progression was not found to be significant (*p* = 0.305); however, no recurrences were observed in 13 patients who received BED_10_ doses above 51.3 Gy. Hepatocellular carcinoma was found to be significantly associated with worse PFS compared to other primaries (*p* = 0.002), which remained significant in multivariate analysis (*p* = 0.022).

### Toxicity

No grade > 3 acute toxicity was observed in any patient. Two patients had grade 2 esophagitis. The patients with esophagitis were those who had also received radiotherapy to the lung previously.

## Discussion

The spine is among the most frequent sites of metastasis, often presenting with symptoms that prioritize local ablative treatments. Conventional palliative radiotherapy for spinal metastases was the standard of care for a long time. However, it was associated with poor 1‑year local control rates and complete pain control, with rates ranging from 61% to 86% and 0% to 24%, respectively, for de novo metastases [10, 11]. Recent developments in the care of cancer patients have led to longer overall survival and necessitated improvements in local control rates and quality of life. Particularly in the oligometastatic setting, where survival rates are higher compared to those in polymetastatic disease, patients may benefit more from aggressive approaches [12]. Spinal SBRT has demonstrated superior local control and complete pain control rates ranging from 57% to 95% and 46% to 92%, respectively [10], and for single-fraction treatments, PTV D_min_ > 15 Gy was shown to improve LC [13]. Consequently, for spinal oligometastases, the treatment paradigm has shifted from palliative conventional radiotherapy to SBRT in recent years.

As high doses with steep dose gradients are delivered with SBRT, protection of organs at risks, especially of the spinal cord, is essential. In recent years, spinal cord dose constraints have been well studied [10]. However, accurate delineation and interfractional imaging of the spinal cord remain challenging. The delineation of spinal cord is ideally done with the fusion of MRI and the simulation CT [14, 15], since MRI has better soft tissue contrast than CT [16]. The use of image-guided radiotherapy, preferably every fraction, is also generally recommended in SBRT for spinal bone metastases [17] to ensure target volume coverage as well as spinal cord sparing.

The MR-linac system allows for online adaptive planning during each treatment fraction. Moreover, it has a double-stack and double-focus multileaf collimator system which allows us to use a steep dose gradient [18]. Also, SMART permits direct visualization of the spinal cord, esophagus, sacral plexus, and other OARs before each fraction, thus enabling contouring of target volumes and organs at risk to evaluate the predicted plan and generate an online adaptive plan. Small setup positional changes can be compensated with adaptive planning in lieu of correcting patient position, which might be hard for patients who have pain due to vertebral metastases. MRI acquisition and on-table adaptive planning may result in longer treatment times with SMART than with cone-beam CT (CBCT)-based systems. Our median treatment time was 15.13 min, which is tolerable given that the repositioning would not be needed due to daily contouring and adaptive planning. Intrafractional motion may also affect the treatment. In their study, Oztek et al. showed that even intrafractional spinal cord motion may contribute to increased spinal cord doses [6]. Real-time tracking with the MR-linac can aid in detecting intrafractional motion and help to prevent both target misalignment and spinal cord overdosing. Our postoperative cases are also a demonstrative example of how 0.35‑T low-strength magnetic field MR images are not severely affected by metallic artifacts, which might be the case for higher magnetic fields which are more susceptible to field inhomogeneities that result in an obscured view of nearby soft tissue structures [19]. Although metallic-artifact-reduction techniques have been developed for CT, no standard technique was developed, and the existing techniques may cause secondary artifacts [20].

Yadav et al. conducted a dosimetric study comparing SBRT performed by MR-linac vs. TrueBeam^TM^ STx-based volumetric modulated arc therapy (VMAT) plans [18]. They evaluated 10 previously treated metastatic lesions. The results showed that SMART plans had lower intermediate dose spillage; similar D_98%_, D_2%_, and D_50%_; and also similar homogeneity and conformity indexes as well as similar doses to the spinal cord, which led the authors to conclude that spinal SBRT delivered with an MR-guided system is comparable to TrueBeam VMAT in terms of target coverage, plan quality, and spinal cord sparing. However, the MR-linac has advantages of contouring the spinal cord and other OARs in every fraction, personalized adaptive online treatment planning, and continuous cine-MR tracking during treatment.

Choi et al. retrospectively generated three plans (MR-linac IMRT, MR-Co-60-IMRT, and VMAT) for 20 thoracic spine lesions with prescription doses of 18 Gy in a single fraction to compare dose–volume outcomes [21]. A notable result was that the average spinal cord volumes delineated based on CT and MRI images significantly varied. Also, while dose de-escalation had to be performed for spinal cord dose tolerances for VMAT and MR-Co-60-IMRT plans, 18 Gy in single fraction could be delivered without dose violation for MR-linac IMRT plans. Importantly, MR-linac IMRT plans resulted in the lowest spinal cord doses and the difference was significant (all *p* ≤ 0.003). Those results accentuate the importance of the MRI-based spinal cord contours as well as the superiority of MR-linac plans both in terms of deliverability of the intended doses and in terms of spinal cord protection.

An ongoing pilot clinical trial is investigating the feasibility of MRI-based simulation and SMART in spinal metastasis with a treatment scheme of five fractions delivered over 1–2 weeks (NCT03878485). Patients with previous radiotherapy to the target region will be excluded. The findings from an ongoing phase I/II clinical trial that utilizes SMART to treat all disease sites, including the spine, and evaluates the feasibility and efficacy of SMART are anticipated (NCT04115254).

Our retrospective results highlight the importance of the aforementioned studies. The study includes real-world data of patients treated with SMART for spinal metastases. Both oligometastatic and palliative irradiations were included, as well as reirradiations, which are not mentioned in the previous literature. We demonstrate that online adaptive plans which may compensate for target motion yield significantly better results in terms of target coverage compared to predicted plans. Spinal cord protection is also significantly improved, and this improvement is even more important for this patient group who are oligometastatic and who may require reirradiation for spinal metastases in the future.

Limitations of our study are its retrospective nature and the fact that some information might be missed, since the dataset was not designed for a prospective study and included very heterogenous patients with different pathologies. Increased follow-up time could yield more comprehensive results.

## Conclusion

Stereotactic magnetic resonance-guided adaptive radiotherapy (SMART) to spinal bone metastases is feasible, and adaptive planning yields improved target coverage and reduced spinal cord doses, which translate into an efficient and safe method to deliver SBRT. It remains unclear whether these dosimetric advantages will be clinically meaningful as compared to CBCT-based spinal SBRT. Further prospective studies are warranted to establish the long-term clinical benefits of this novel approach.

## Data Availability

Research data are stored in an institutional repository and will be shared upon request to the corresponding author.
